# P-249. Healthcare Utilization and Costs in People Living with HIV and Neuropsychiatric Disorders in the United States

**DOI:** 10.1093/ofid/ofaf695.471

**Published:** 2026-01-11

**Authors:** Sean P Fleming, Shweta Kamat, Girish Prajapati, Viktor Chirikov, Wenying Quan, Mark Bounthavong

**Affiliations:** Merck & Co., Inc., Rahway, NJ, USA, Philadelphia, Pennsylvania; OPEN Health Evidence & Access, New York, New York; Merck & Co., Inc., Rahway, NJ; OPEN Health Evidence & Access, New York, New York; OPEN Health Evidence & Access, New York, New York; University of California, San Diego, La Jolla, California

## Abstract

**Background:**

People living with HIV (PLWH) have increased prevalence and risk of neuropsychiatric disorders. This study assessed incremental all-cause healthcare resource utilization (HCRU) and costs among US PLWH with vs. without neuropsychiatric disorders.

**Figure:**
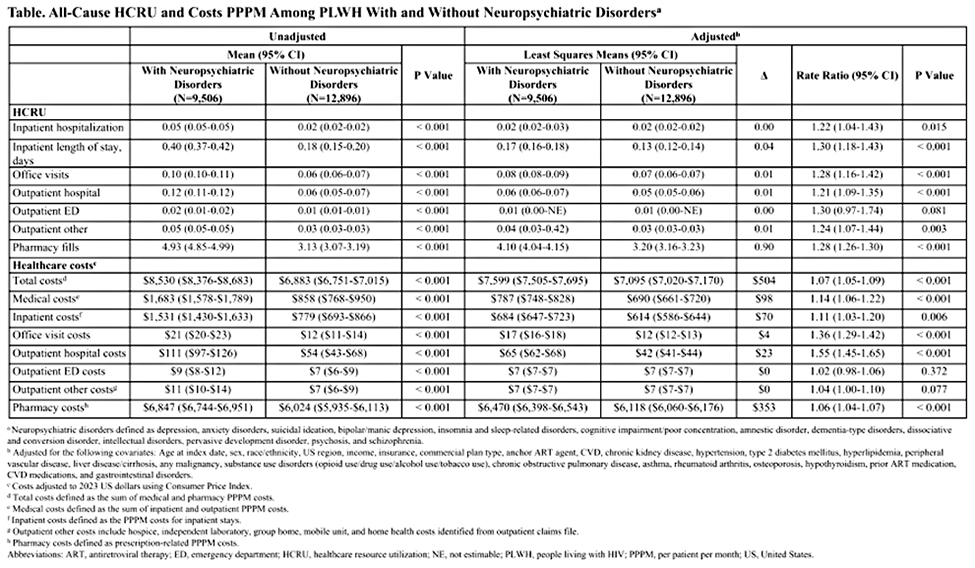

**Methods:**

A retrospective analysis of US administrative claims (Jan 2020-Dec 2022, Optum’s de-identified Clinformatics® Data Mart Database) examined all-cause HCRU and costs among adult (≥ 18 years) PLWH with ≥ 1 pharmacy claim for anchor antiretroviral therapy (ART) agent (NNRTI, PI, or INSTI) in 2021 (index date: earliest anchor ART claim). PLWH were followed to the earliest of 12 months or end of continuous enrollment and stratified into 2 groups based on the presence of neuropsychiatric disorders (yes/no) during baseline (12 months pre-index) using *ICD-10* diagnosis codes from medical claims. Differences in all-cause per-patient-per-month (PPPM) HCRU and costs (converted to 2023 USD) between groups were estimated using multivariable generalized linear models with negative binomial/Poisson distribution (HCRU) and gamma distribution (costs), adjusting for baseline characteristics.

**Results:**

Of 22,402 PLWH identified, 9,506 (42%) had neuropsychiatric disorders. PLWH with vs. without neuropsychiatric disorders were older (mean age 56 vs. 54 years), more were women (21% vs. 17%) and White (51% vs. 44%), and had higher mean Quan-Charlson Comorbidity Index scores (1.82 vs. 0.92) and baseline total costs ($4,579 vs. $3,416); all p < 0.001. Unadjusted all-cause PPPM HCRU and costs were significantly higher in PLWH with vs. without neuropsychiatric disorders (all p < 0.001; Table). In multivariable analyses, PLWH with vs. without neuropsychiatric disorders generally had significantly greater all-cause PPPM HCRU, including office visits (28% higher), and costs, but outpatient ED visits/costs and outpatient other costs were not significantly higher (Table).

**Conclusion:**

PLWH with neuropsychiatric disorders experience a greater HCRU and cost burden than those without neuropsychiatric disorders. Further investigation of the role of HIV, including ART, in the development of neuropsychiatric disorders may help improve individualization of care and prevent excess HCRU and costs.

**Disclosures:**

Sean P. Fleming, PhD, MSW, Merck & Co., Inc., Rahway, NJ, USA: Employee|Merck & Co., Inc., Rahway, NJ, USA: Stocks/Bonds (Public Company) Shweta Kamat, MS, PhD, Merck & Co., Inc., Rahway, NJ, USA: Contracted research Girish Prajapati, M.B.B.S., MPH , Merck & Co., Inc.: Employee|Merck & Co., Inc.: Stocks/Bonds (Private Company) Viktor Chirikov, MS, PhD, Merck & Co., Inc., Rahway, NJ, USA: Contracted research Wenying Quan, MS, Merck & Co., Inc., Rahway, NJ, USA: Contracted research Mark Bounthavong, PharmD, PhD, Merck & Co., Inc., Rahway, NJ, USA: Consultant|University of California, San Diego: Employment

